# Internet-based surveillance to track trends in seasonal allergies across the United States

**DOI:** 10.1093/pnasnexus/pgae430

**Published:** 2024-10-29

**Authors:** Elias Stallard-Olivera, Noah Fierer

**Affiliations:** Cooperative Institute for Research in Environmental Sciences, University of Colorado, Boulder, CO 80309, USA; Department of Ecology and Evolutionary Biology, University of Colorado, Boulder, CO 80309, USA; Cooperative Institute for Research in Environmental Sciences, University of Colorado, Boulder, CO 80309, USA; Department of Ecology and Evolutionary Biology, University of Colorado, Boulder, CO 80309, USA

**Keywords:** seasonal allergies, social media, natural language processing, internet-based disease surveillance

## Abstract

Over a quarter of adults in the United States suffer from seasonal allergies, yet the broader spatiotemporal patterns in seasonal allergy trends remain poorly resolved. This knowledge gap persists due to difficulties in quantifying allergies as symptoms are seldom severe enough to warrant hospital visits. We show that we can use machine learning to extract relevant data from Twitter posts and Google searches to examine population-level trends in seasonal allergies at high spatial and temporal resolution, validating the approach against hospital record data obtained from selected counties in California, United States. After showing that internet-derived data can be used as a proxy for aeroallergen exposures, we demonstrate the utility of our approach by mapping seasonal allergy-related online activity across the 144 most populous US counties at daily time steps over an 8-year period, highlighting the spatial and temporal dynamics in allergy trends across the continental United States.

**Significance Statement**

A substantial portion of the US population suffers from seasonal allergies, but obtaining robust estimates of when and where seasonal allergies are highest has been challenging. We demonstrate that internet-derived data, combined with machine learning and advanced statistics, can quantify spatial and temporal patterns in seasonal allergies. We also show that multiple internet-based methods can be used as proxy measures to obtain similar results, an issue that impacts researchers relying on internet data sources as online habits change over time. As the timing and magnitude of seasonal allergies are expected to shift with ongoing land-use and climate change, the approach we have developed and validated will be increasingly valuable for predictive modeling of seasonal allergies and aeroallergen exposures.

## Introduction

Allergic diseases—which include atopic asthma, atopic conjunctivitis, atopic dermatitis, and allergic rhinitis—are estimated to affect approximately 26% of the American population (1) and to cost the United States economy an estimated $4.5 billion to more than $40 billion dollars per year in direct and indirect costs (2–4). Not only do these conditions reduce quality of life and long-term health outcomes, seasonal allergies can also significantly reduce the productivity of workers in both physically and nonphysically demanding jobs (5, 6). Moreover, seasonal allergies are expected to worsen with climate change-induced increases in aerial mold and pollen exposures (7–10), but such changes in aeroallergen exposures remain difficult to quantify.

Despite the broader relevance of seasonal allergies to public health, the spatiotemporal patterns in allergic disease remain poorly understood. This is largely because the majority of cases initiated by seasonal allergies are not sufficiently severe to warrant visits to clinics or hospitals where they would be documented. This means that formal medical record analyses typically fail to capture spatiotemporal changes in how seasonal allergies are trending across large geographic regions at reasonably high resolution.

Due to this absence of accessible population-level surveillance data on seasonal allergies, researchers have had to rely on alternative strategies to assess allergic disease in a population. These include relying on self-reported data (11) or assuming a constant relationship between seasonal allergy symptoms across a population and concentrations of airborne pollen or other aeroallergens (12, 13). While these approaches can be useful, there are important limitations. Self-reporting of allergy symptoms is based on opt-in programs requiring the use of dedicated smartphone apps and websites, which can limit the pool of participants (11). Likewise, predicting trends in seasonal allergies from measured or inferred aeroallergen concentrations can be restrictive given that the available data on aeroallergen exposures is often only available for specific locations or limited to pollen alone (12, 14), resulting in poor spatial resolution. This constraint is particularly evident in the United States where aeroallergen monitoring networks are less well developed as compared to those found in the European Union and Japan (12). We also know that the specific triggers of seasonal allergies can be highly variable. Seasonal allergies can be caused by pollen released from trees, grasses, and weeds, as well as exposure to airborne fungi (molds) and bacterial endotoxins (3, 15) with the relative importance of the specific triggers of seasonal allergies varying across time and space in ways that can be difficult to determine a priori (16).

We propose that internet-based surveillance could serve as an alternate approach to track seasonal allergies across the United States. Internet-based surveillance has been widely used over the last 15 years as a tool to understand the spread of disease and for predicting outbreaks through text analysis and volunteered geographic information on platforms such as Twitter, Facebook, Google, Instagram, and Yelp. Such approaches have been applied to a number of different types of diseases, with models built for tracking dengue, influenza, food poisoning, COVID-19, and Ebola as examples (17–20).

While internet-based disease surveillance shows promise from a technical perspective, there have been problems with its accuracy and applicability, for example the widely publicized failure of Google Flu Trends to predict flu hospitalizations despite years of development (21). Due to these problems, attention is being directed towards making internet-based disease surveillance more effective. Importantly, the integration of internet-based models with traditional surveillance methods (22) is key to gauging the efficacy of internet-based approaches. Our study builds on previous studies that have used internet-based surveillance to infer trends in seasonal allergies across a population (23–26), improving the approach by validating it against public health data and demonstrating its utility for estimating allergy dynamics across broad spatial scales over multiple years at high temporal resolution.

We hypothesize there is a notable statistical relationship between hospital record data and allergy-related internet activity, suggesting the potential for internet-based surveillance to serve as an indicative proxy of population-level trends in allergic diseases. If our hypothesis is valid, it could enable the observation of trends in seasonal allergies across different geographic regions with improved spatiotemporal resolution compared to current methods.

In designing longitudinal studies that harness social media data, it is crucial to anticipate potential changes in any given internet platform's format, ownership, or popularity (22). Thus, to test our hypothesis, we accessed historical search patterns from multiple digital data sources—Google Trends and posts from Twitter's archive—using machine learning to identify and quantify patterns in allergy-related internet activity over time and across space. These data were validated by comparison to daily hospital record data, specifically emergency department (ED) visits coded for allergic rhinitis, atopic dermatitis, or atopic conjunctivitis for each county in the state of California over a 4-year period from 2016 through 2019 to determine the empirical relationship between the variables, assuming that allergy-related Twitter posts and Google searches are a reliable indicator of people's self-perception of allergy symptoms in the general population. We showed that there is a cointegrating relationship in the spread between internet data and allergy ED coding records, as well as correlation between the raw and residual time series of the cointegrated time series. After showing that we can effectively capture spatiotemporal trends in allergic disease across the more populous California counties (those with >500,000 people, 16 counties in total), we then demonstrate how our model can also be used to estimate population trends in allergic disease in 144 counties (each with >500,000 people) across the continental United States at daily resolution over an 8-year period.

## Results and discussion

### Cointegration of ED visit data, Twitter posts, and Google search frequency

Our first objective was to determine if online activity could serve as a reliable proxy for trends in seasonal allergies. Thus, we started by comparing the occurrence of allergy-related Twitter posts and Google searches from California with hospital record data from the state to validate our online activity-based approach before applying our approach nationwide. To do so, we performed cointegration tests to identify long-term equilibria and common trends, and cross-correlation tests for short-term linkages, with adjustments for seasonality and trends.

**Fig. 1.**
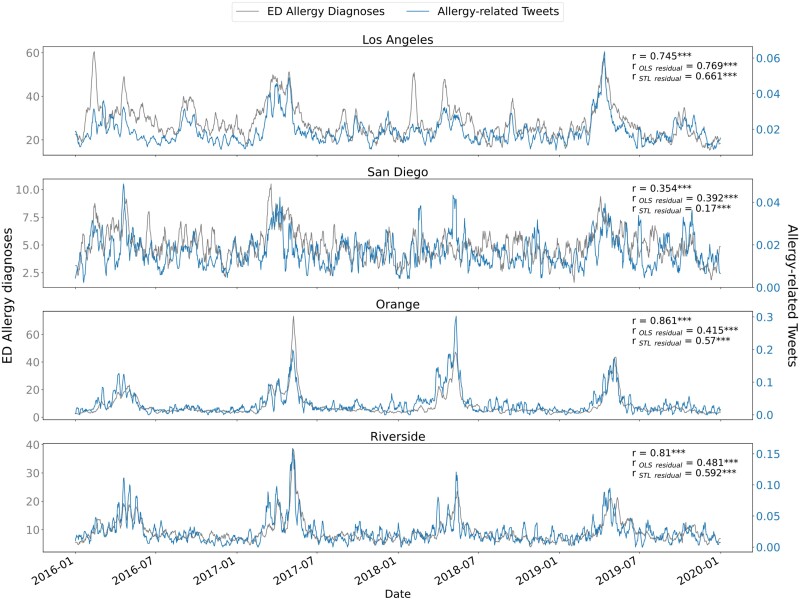
Allergy-related ED visits vs. allergy-related twitter posts, 7-day rolling average. Temporal trends in raw allergy-related Twitter posts and allergy-related ED records in four California counties (see Fig. S1 for results from all counties used for validation of our approach). The Pearson correlation coefficients for both raw and residual values are included for comparison against the raw correlations.

Since time series with trends or deterministic seasonality are inherently nonstationary, we needed to process the time series under investigation before standard modeling and estimation procedures could be used. To determine if correlation testing is a valid method for identifying linear relationships between these time series, we first checked for cointegration between ED records and either allergy-related Twitter posts (Figs. 1 and S1) or Google searches (Fig. S2), given the importance of cointegration testing for handling these types of time series data (27–31). The results of the cointegration tests show that Twitter posts (Table S1) and Google searches (Table S2) are cointegrated with ED records as well as with each other (Fig. S3) across the majority of counties and Designated Market Areas (DMAs) in California, which were the smallest spatial units available from Google Trends. These cointegration results indicate that, despite potential biases in sample populations, these three datasets share a long-term equilibrium and that the spread between internet-based surveillance methods and traditional surveillance methods is stationary (Tables S1 and S2). As cointegration does not imply a causal relationship, and we have no reason to believe that ED records have a causal relationship with either Twitter posts or Google searches, we can conclude that there is an unobserved external factor that ties these variables into a cointegrating relationship.

Given there is an accepted relationship between exposure to aeroallergens and allergic disease (15), this external factor is likely airborne concentration of ecological allergens (namely pollens and molds). Furthermore, the correlation between both the raw and residual Twitter (Table S1) and Google (Table S2) data with ED records suggests that anomalies in the data could serve as a foundation for exploring specific aeroallergen regimes that disproportionately influence the incidence of allergic diseases at a particular time and place (32).

### Correlation between ED visit data, Twitter posts, and Google search frequency

The cross-correlation findings demonstrate reasonably strong and statistically significant linear relationships between online activity pertaining to seasonal allergies and ED records (Tables S1 and S2). These results suggest that these elements not only share a long-term equilibrium, as indicated by their cointegration, but they also align closely in most cases (Fig. S3). Since the time series are cointegrated (see above), we can calculate a zero-lag cross-correlation between the raw and residual time series—equivalent to a Pearson correlation—without risking spurious results. We also examined both parametric and nonparametric residuals, checking for a linear relationship that could potentially be used for an analysis of anomalies and short-term trends. The calculated Pearson correlations of these residuals were generally greater than 0.5 in the more populous California counties, further reinforcing the significant time-independent relationship between allergy-related Twitter posts or Google searches and ED records (Table S1). In counties with fewer than 450 allergy-coded encounters per 100,000 people or fewer than 80 Twitter posts per 100,000 people, available ED record data and Twitter post volume were frequently too low to assure data quality resulting in a wide range of correlation values (Fig. S6 and Table S1). Counties exceeding these thresholds are typically home to more than 500,000 people. For this reason, all downstream analyses (including the US wide analyses, see below) were restricted to counties with populations >500,000. Together, our results show that online activity is not only cointegrated with hospital data, indicating a stable long-term relationship, but also exhibits significant short-term correlation in the more populous counties of California, providing the statistical validation to justify our use of online activity for monitoring seasonal allergy trends across the United States. However, it must be noted that correlation will be impacted by biases in the sample populations, limiting the usefulness of this strategy for predictions of population means or for implementing healthcare interventions.

### Nationwide analyses

After validating our Twitter-based model against medical record data from California as described above, we then used our model to infer seasonal allergy patterns across the entire United States using posts from 2016–2022 across 144 counties that have populations over 500,000 as per the 2020 census. We want to reiterate that we can only infer seasonal allergy patterns for counties with >500,000 people as the comparison of our Twitter-based model against California hospital record data (described above) shows that less populous counties do not typically have enough geolocated Twitter data to form a dense time series. While these limitations mean that this approach cannot capture trends in less populous regions of the United States, these 144 counties (each having populations over 500,000) collectively represent over half the US population.

**Fig. 2.**
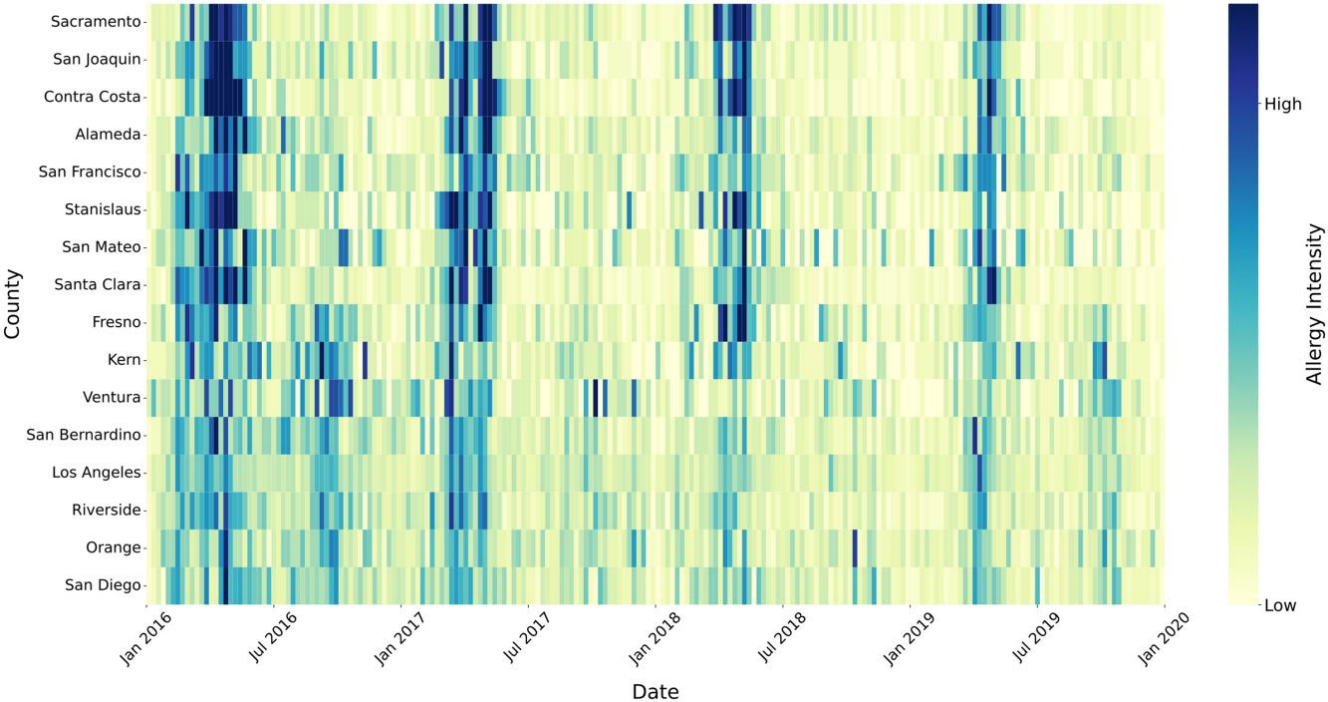
Annualized allergy intensity by county averaged across 2016–2022. Z-scores indicate a relatively high intensity of yearly allergies on the East Coast, with lower intensity allergies in Florida and Southern California. The counties with the highest annual Z-scores, indicating countries experiencing the most severe annualized allergies over the 8-year period, are located in the southeastern United States. Other regions with moderately high allergy intensities are in California's Northern Central Valley as well as the Mid-Atlantic region.

**Fig. 3.**
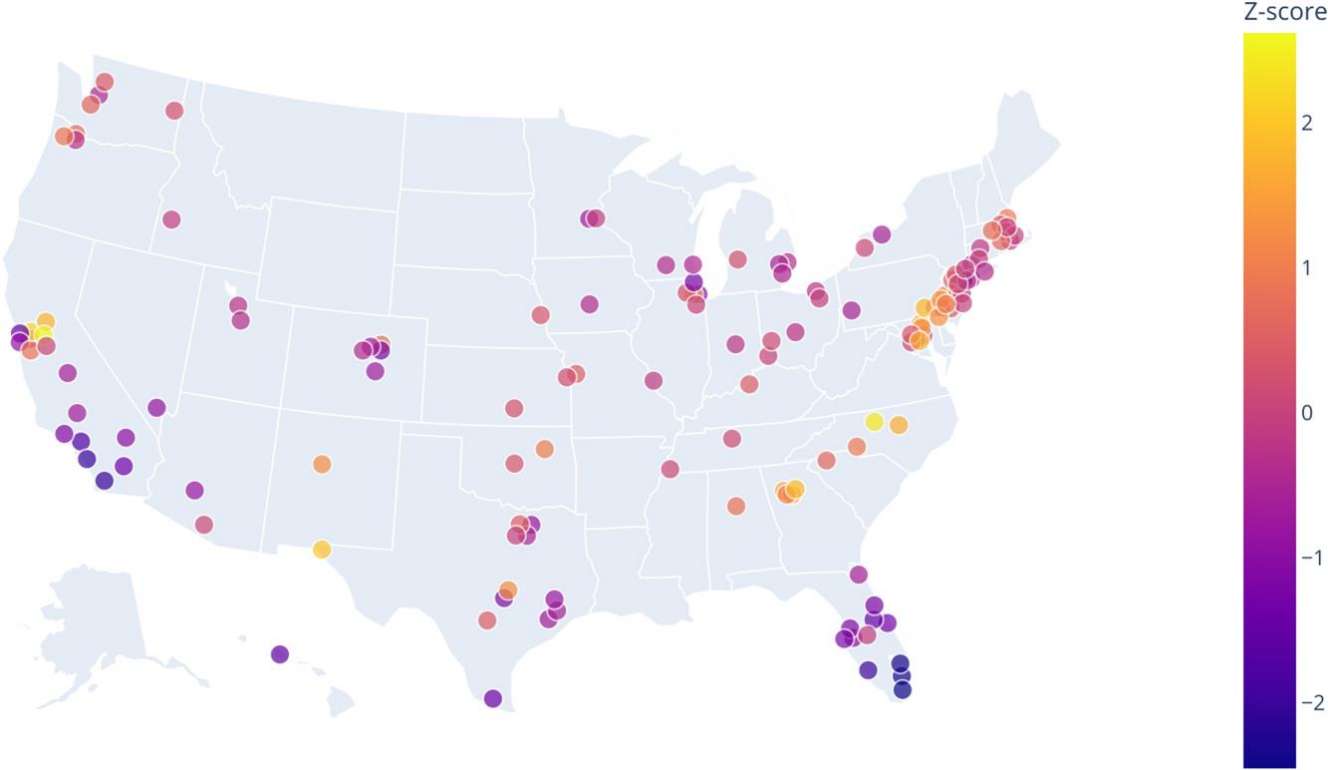
Seasonal patterns in allergy intensity across the 144 counties in the United States with a population above 500,000, based on the Z-score of Twitter post volume averaged across 2016–2022 related to seasonal allergies. Spring months (March–May) represent the dominant allergy season and fall months (September–October) represent the second most prominent season; summer and winter allergy seasons are less pronounced but can be important in specific regions (including Colorado, Florida, and Texas).

This nationwide extension of our Twitter-based model reveals that seasonal allergy intensity varies widely across the United States (Fig. 2). Nearly every county we examined demonstrates a fairly substantial spring allergy season (March–May), with secondary peaks appearing during the fall allergy season (September–October). Winter and summer allergy seasons appear in certain parts of the country (including the Mountain West, Texas, and Florida), but allergy intensity was typically low across most of the United States during these seasons (Fig. 3). The timing and characteristics of allergy seasons can, however, vary between neighboring counties, possibly due to local climatic factors or differences in aeroallergen sources. For example, the peak of spring allergy season occurs earlier in Central Valley counties of Northern California than in the Bay Area despite their close proximity (Fig. 2).

We also observed a latitudinal temporal gradient from north to south across the continental United States that corresponds to the peak date of the various allergy seasons, with the seasons peaking earlier in more southerly latitudes (Fig. 3) and matching known pollen release patterns (33). The earliest counties to experience spring allergy season are concentrated in the southeastern United States (Fig. 3). In contrast, the last counties to experience spring allergy season are typically in the Northeast and Upper Midwest of the United States (Fig. 3). We also show that the intensity of allergy seasons can vary considerably year-on-year, and there can be outsized increases or decreases in allergy-related Twitter posts over short periods of time which deviate from typical seasonal patterns observed in individual counties (Movie S1).

**Fig. 4.**
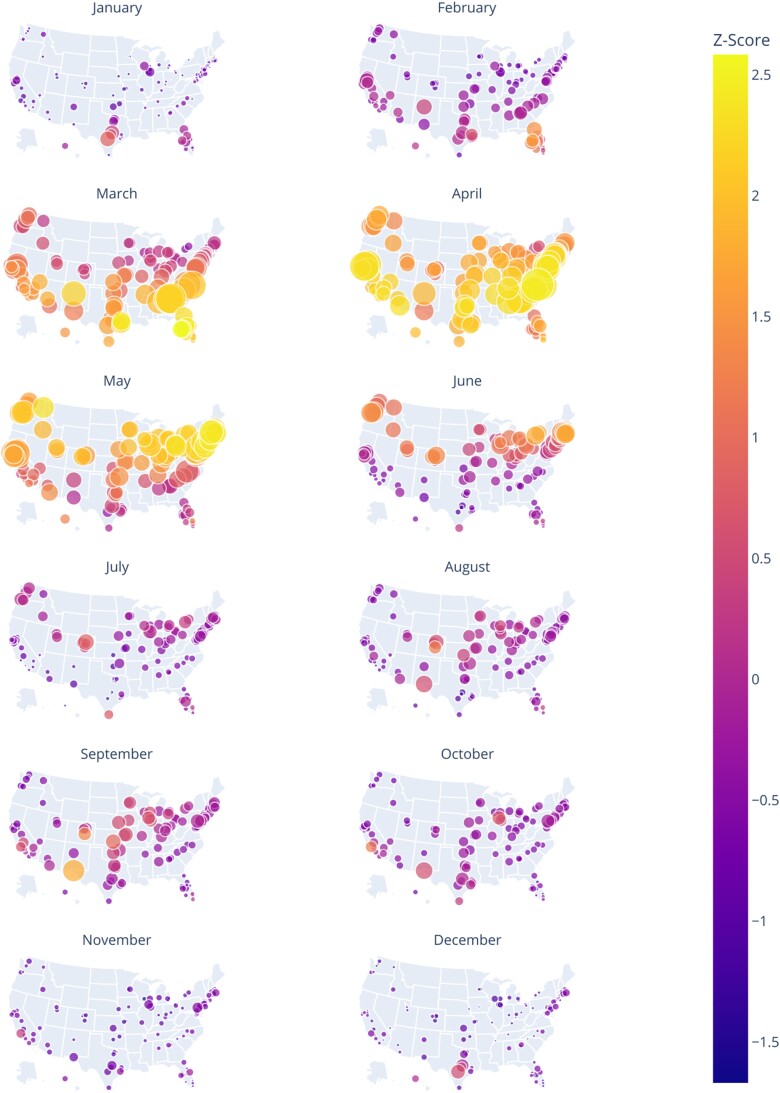
Weekly allergy intensity as measured by the raw probability of allergy-related Twitter posts in California by county, arranged north to south for all counties that had a sufficiently large number of geolocated Twitter data (>500,000 people). Allergy intensity was calculated based on allergy-related Twitter posts generated over a period from 2020 January 1 through 2019 December 31. Allergy intensity can vary substantially between years, with some years having shorter allergy seasons than others, a trend not readily visible in the annualized national data (Fig. 3).

We can also use our Twitter-based model to quantify interannual spatiotemporal trends in the timing and intensity of allergy seasons across geographic regions and between years. For example, a closer analysis of seasonal trends in California shows that not only does the intensity of spring allergy season change between years, but in Southern California the intensity of fall allergy season varies so greatly that it is nearly absent in some years despite the average year showing a relatively strong fall allergy season (Fig. 4). The differences in aeroallergen regimes associated with regional and seasonal differences warrant further investigation. For example, while pollen is considered the main driver of seasonal allergies (34), other factors such as mold and bacterial endotoxins may also play a significant role, especially during fall and winter seasons (35) as well as during agricultural harvest events (36–38).

## Conclusions

We show that internet-based disease surveillance can supplement traditional methods of allergic disease surveillance to enable the rapid, large-scale data collection needed for modeling allergic disease and its ecological drivers. There is a robust relationship between allergy-related online surveillance data and records of allergy-related patient codes submitted in hospital ED. The cointegration of these variables illustrates a shared long-term equilibrium, further substantiated by a linear correlation. After initial validation of our approach, using ED records from California to validate seasonal allergy-related online activity, we expanded our internet-based surveillance using Twitter data to infer allergic disease patterns across the United States from 2015 to 2022. This analysis uncovered unique trends of seasonal allergies across different regions and times in the United States (Fig. 3).

Further exploration of these patterns using climate- and land-use-based models coupled with ground-level measurements of aeroallergen types and concentrations is warranted to pinpoint the specific allergens responsible for allergic diseases. This approach would enable researchers to determine if outliers in the time series, such as that seen in Los Angeles County in June 2018 (Supplements 3, 4), are the result of a general increase in aeroallergen concentrations or if they are the result of higher concentrations of specific aeroallergens (e.g. specific pollens or molds). Long-term tracking of allergy-related online activity could enhance our ability to predict how allergic disease trends may respond to climate change over extended periods, especially when combined with real-time monitoring of aeroallergen loads.

Our methodology, which incorporates both traditional and multiple sources of internet-based surveillance, offers a more robust approach than those dependent solely on a single type of online activity, and could be further improved by adding additional data sources, such as Facebook engagement data (39). Our approach does not presuppose a relationship between an environmental variable, such as temperature or humidity, and seasonal allergies (26). Instead, it complements traditional surveillance in monitoring and potentially predicting allergic diseases. Our validated approach yields useful inferences of allergic disease intensity that are particularly valuable where traditional data sources are scarce. However, this method requires a population with sufficient size and internet use, otherwise the data are too sparse or noisy for accurate results, limiting its applicability to more populous regions. This shortcoming could be overcome by network and semantic analyses to allow our model to be applied to more sparsely populated areas (40). Coupling this with a task-specific text classifier could improve the accuracy of online seasonal allergy tracking and provide a nearly real-time stream of allergy-related social media activity to compare to aeroallergen monitoring data for the improved forecasting of seasonal allergies.

## Materials and methods

### Data acquisition and processing

#### ED record data

International Classification of Disease (ICD) codes are often used in traditional disease surveillance. One use of ICD codes is by healthcare providers to classify patient conditions for recording and billing purposes (41). Starting in 2015, ICD-10 records began including primary and secondary codes for conditions linked to seasonal allergies such as allergic rhinitis, atopic eczema, and atopic conjunctivitis as categories independent of related conditions such as asthma. We requested hospital ED visit records from the California Department of Health Care Access and Information (HCAI) where IDC-10 codes related to allergic disease (Table S3) were recorded as primary or secondary codes from 2016 January 1 through 2020 December 31. We did not request historical ICD-10 code data from before 2016 January 1 as coding at that time was based on ICD-9. These data were provided at a daily temporal resolution and county-level spatial resolution. To adhere to the provisions of the Health Insurance Portability and Accountability Act (HIPAA), data from the least populous 16 counties were masked and excluded. After review, we removed the year 2020 from the dataset as the COVID-19 pandemic altered patterns of hospital attendance (42), limiting the usefulness of ED code data for that year. Other data available from HCAI, including patient discharge data, proved unsuitable for this analysis, and the inclusion of data that would allow for bias correction, namely ethnic and racial data, would have caused this data to be classified as having high potential risk for a HIPAA violation, and as such was not requested.

#### Twitter data

Internet-based surveillance data were acquired from Twitter and Google. To build a dataset of Twitter posts, we examined the most frequent seasonal allergy- and symptom-related words and phrases derived from known allergy-related posts in order to develop a list of terms and hashtags (Table S3) which we expect to find in Twitter posts where posters perceive themselves to be experiencing symptoms associated with seasonal allergies. Twitter posts containing terms matching this list and posted within the state of California were collected for the period from 2016 January 1 through 2020 December 31 via the Twitter for Academic Research application programming interface (API) full-archive search functionality. To relate these posts to specific geographic areas, only geotagged posts were included in the analysis. A total of 67,033 Twitter posts meeting these criteria were downloaded. Only 1–3% of all Twitter posts are geotagged to a specific location, and this selection proved sufficient for data analysis. As seasonal allergies impact such a large portion of the general population, we proceeded under the assumption that the proportion of geotagged users posting about seasonal allergies is proportional to the portion of the general population experiencing seasonal allergies.

For the nationwide analyses, we collected Twitter posts containing the same list of terms and hashtags as the California Twitter dataset, but for the entire continental United States over the period from 2016 January 1 through 2022 December 1. As this dataset was not compared to ED record data, it was not necessary to remove posts made after the beginning of the COVID-19 pandemic as the classification model was trained to remove posts related to COVID-19.

#### Twitter data processing

Like most other internet-based surveillance methods, Twitter surveillance is dependent on machine learning for accurately identifying relevant posts, and there is now robust evidence that neural networks for natural language processing can be used with text embeddings to identify Twitter posts of interest for researchers (24, 43–46). Accurate post-identification is important as many Twitter posts containing the target search terms do not actually relate to seasonal allergies. For example, posts containing the words “sneeze” and “allergies,” such as “Tis the season to be sneezing up a storm” and “My allergies are really bad today!!,” indicate that the user was experiencing seasonal allergies while posts like “@BabyAnimalPics: a chicken sneezing…. that is all” and “Gluten and Allergy Free expo” do not. Removing irrelevant Twitter posts necessitates the creation of an annotated dataset and automated text classifier to identify and categorize the posts as either relevant or irrelevant.

To build the annotated test and training set, a random subset of 4,000 Twitter posts from the national dataset was manually labeled as relevant or irrelevant in a simplification of the training method proposed previously for text classification-based identification of allergy-related Twitter posts (26). A natural language processing model based on Bidirectional Encoder Representations from Transformers was then used to classify the Twitter posts according to the labels as per the previously described methods (47–49). We selected the pretrained roBerta-base model classifier (50) as the basis for fine-tuning our model to specifically identify seasonal allergy-related Twitter posts. The tuned classifier achieved an accuracy of 0.88, a precision of 0.94, and a recall of 0.86 on the dataset after training. The tuned classifier was then run on the remaining 63,033 posts and all Twitter posts labeled as irrelevant by the classifier were removed from the dataset, resulting in a total of 42,868 relevant posts being considered for further analysis.

Twitter assigns geotagged posts a bounding box associated with a city, neighborhood, or point of interest alongside a specific timestamp. To build a time series of posts that matches the resolution of the county-level HCAI data, posts were aggregated by day and the centroids of the bounding boxes of each post was assigned to the specific county containing those coordinates with PostGIS (51, 52). These efforts ultimately resulted in a dataset containing a time series for each county in California measuring daily allergy-related tweet counts.

#### Google trends data

A secondary dataset was constructed based on the daily probability that terms related to seasonal allergies were searched for on Google. The list of terms was based on a list of keywords and Freebase ID codes, which are unique identifiers developed by Metaweb Technologies and utilized by Google Trends to classify topics and entities that can be related to searches. We examined codes related to seasonal allergies, antihistamine medications, environmental allergy symptoms, and search topics categorized as being associated with people seeking information on seasonal allergies. These data were downloaded from Google's private Google Extended Trends for Health (GT-E) API, formerly Google Flu Trends.

GT-E data are based on a random sample of 10–15% of Google searches, with an additional subset sampled and updated daily. To develop a representative time series of searches, it is necessary to sample GT-E data corresponding to the same terms and time period multiple times, as per previously published recommendations (53). In the United States, GT-E data are available at state and Nielsen designated market area (DMA) spatial resolutions. DMAs are areas used by Nielsen to measure local television consumption and represent the smallest geographic resolution offered by GT-E; however, DMAs are typically larger than counties. With the exception of the Palm Springs and Bakersfield DMAs, counties can be mapped directly to DMAs in California enabling a comparison of county-level and DMA-level data. Most DMAs map to multiple counties, and so to directly compare GT-E data to Twitter and ED code data, the latter two were aggregated into their respective DMAs, resulting in a second dataset of daily DMA-level allergy-related tweet counts and ED counts coding records for the purpose of direct comparison to GT-E data.

#### Denoising, quality control, and reconciliation of geographic data

Due to the noise inherent in low volume samples from large populations, a 7-day moving average was taken of the allergy-related Twitter, Google, and ED record data. This served as a low-pass filter to remove high-frequency noise and eliminate periodic weekly effects. Many of the keywords and Freebase IDs selected for allergy-related Google Trends analysis also proved to be of low value, with most showing no clear temporal patterns. The Freebase ID code “/m/0cnq0,” which corresponds to the Freebase ID topic of “allergic rhinitis,” was selected as best representing searches relating to seasonal allergies.

#### Caveats and biases in the data

The data utilized in this study present several caveats and biases that we want to carefully acknowledge. First, none of the data inputs are derived from probabilistic samples, making calculation of mean population levels of allergic disease difficult. The Google Trends Extended for Health API (GT-E), while based on estimates from randomly sampled Google Search users, does not necessarily represent the general population as different demographics exhibit varying search tendencies. Additionally, the HCAI's ED encounter data is an administrative sample, with a skewed representation towards individuals less likely to possess private insurance (54). Twitter data also present challenges as it is likely skewed towards younger individuals and those with higher socioeconomic status (55); furthermore, those with geotagging enabled show additional deviation from the general Twitter demographic (56). Finally, the demographics of those who suffer from seasonal allergies are also different from the general population (1). Despite these biases, the primary objective of this project was to examine the relationships between trends in the data, aiming to assess if online activity can serve as a reliable proxy for tracking trends in aeroallergen exposures across a population in the long term through co-movements.

### Nationwide data processing

#### Nationwide Twitter data

To demonstrate the applicability of internet-based surveillance for seasonal allergy mapping, Twitter data were downloaded for every county in the continental United States between 2016 January 1 and 2022 December 1. These data were processed following the methods described above. Similar thresholds for consideration were applied to the nationwide counties, with all counties having fewer than 500,000 inhabitants excluded from the analyses, yielding a total of 476,094 posts, of which 290,796 were allergy-related. To convert the raw post count into the probability that allergy-related posts are being made on any given day, the number of allergy-related posts for each were aggregated by day and county using the same technique as in the California dataset and divided by the total number of posts for each Twitter-assigned place which were also aggregated by day and county. GT-E data were not considered for the national analyses as the DMA-level resolution of the data means that areal units can be as large as entire US states, limiting their usefulness in examining localized and cross-regional trends.

### Statistical analyses

#### Correlation testing

Correlations between online activity and allergy-related ED visits were calculated by taking the Pearson correlation between their respective time series, aggregated at day and county level. To investigate events out of equilibrium, Pearson correlations of both parametric and nonparametric residuals were also calculated from ordinary least-squares regression of the time series with seasonal dummy variables and seasonal-trend decomposition with locally estimated scatterplot smoothing, respectively, further demonstrating the significant time-independent relationship between allergy-related Twitter posts or Google searches and ED records by day and county (Figs. S4 and S5). Stationarity of the decomposed residuals was verified with the Augmented-Dicky Fuller test.

#### Cointegration testing

To underscore the presence of a seasonal unit root in each variable for the various counties and DMAs, we employed the Canova-Hansen test for seasonal stability (57). Our findings confirm that every single California county with a population exceeding 500,000 and all DMAs exhibit a seasonal unit root, with a differencing order of 1 (Tables S1 and S2). For Twitter data, two-step Engle-Granger tests for cointegration were performed between the daily time series of Twitter posts and ED records for each county twice, with each series regressed against the other, in order to verify cointegration in both directions. The Engle-Granger tests demonstrated that even in counties with less frequent allergy-related tweeting—generally low-population counties—the long-term equilibrium between allergy-related Twitter posting and ED coding is strong enough to see cointegration at a 1% critical value (Table S1). However, the lack of Twitter posts observed for stretches of time in low-population counties makes the data in those counties questionable (see Results). For Google search data, the Johanssen-procedure was performed in R using the urca package (56) with Twitter posts, ED codes, and Google search data for each DMA simultaneously, demonstrating that all three time series are also collectively cointegrated at the DMA level with similar results to the county-level data (Table S2), implying commutability between internet-based surveillance methods.

## Supplementary Material

pgae430_Supplementary_Data

## Data Availability

All aggregated and de-identified data and analysis code used for this study are available at https://zenodo.org/doi/10.5281/zenodo.12741286.
