# Supplementary material for: Internet-based surveillance to track trends in seasonal allergies across the United States
Source: PNAS Nexus. 2024 Oct 29;3(10):pgae430. doi: 10.1093/pnasnexus/pgae430 (PMC11521329; doi:10.1093/pnasnexus/pgae430)
Supplement: pgae430_Supplementary_Data [file pgae430_supplementary_data.zip › PNASNEXUS-PNASNEXUS-2023-01396-TR-s02.docx]

**Supporting Information for**

Internet-based surveillance to track trends in seasonal allergies across the United States

**Authors:** Elias Stallard-Olivera, Noah Fierer

Elias Stallard-Olivera
**Email:**  elias.stallardolivera@gmail.com

**This PDF file includes:**

Supporting text

Figures S1 to S6

Tables S1 to S3

Legends for Movie S1

**Other supporting materials for this manuscript include the following:**

Movies S1


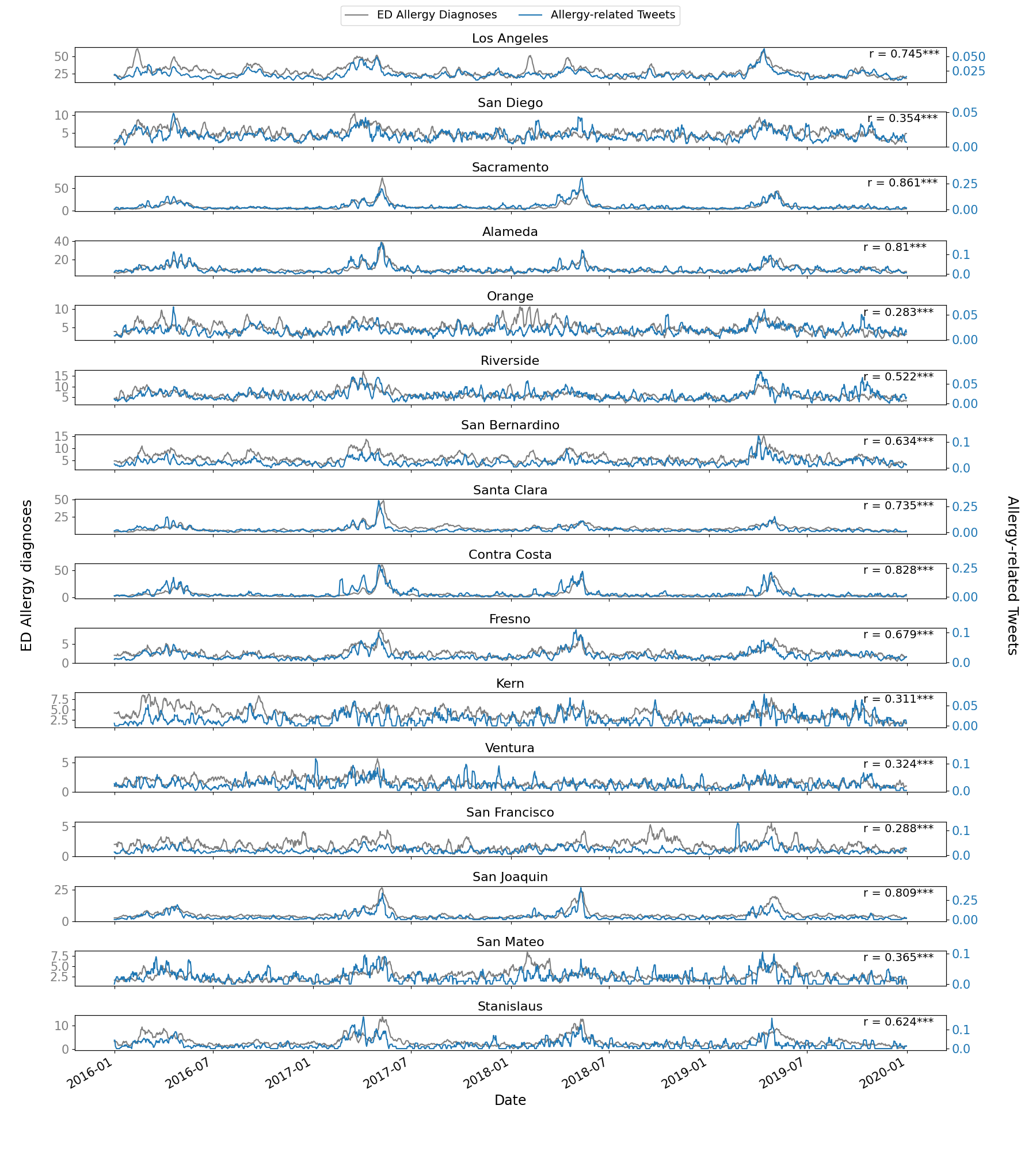
**Fig. S1: Temporal trends in allergy-related ED visits and allergy-related Google search activity across the 16 most populous California counties.** The Pearson correlation coefficient for raw values is included.


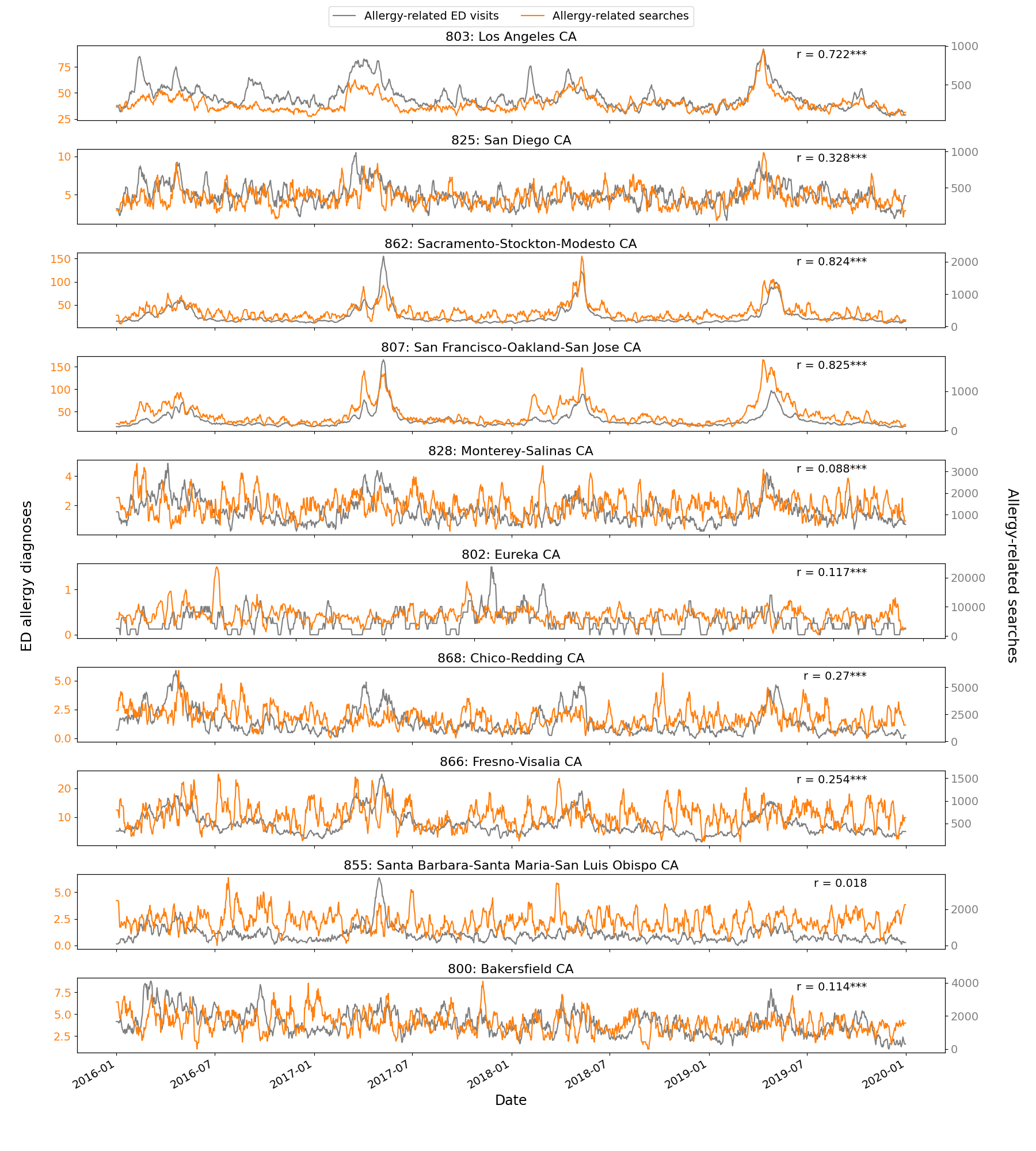


**Fig. S2: Temporal trends in allergy-related ED visits and allergy-related Google search activity across 10 California DMAs.** The Pearson correlation coefficient for raw values is included. Low population DMAs include 802: Eureka, 868: Chico-Redding, 828: Monterey-Salinas, and 855: Santa Barbara.


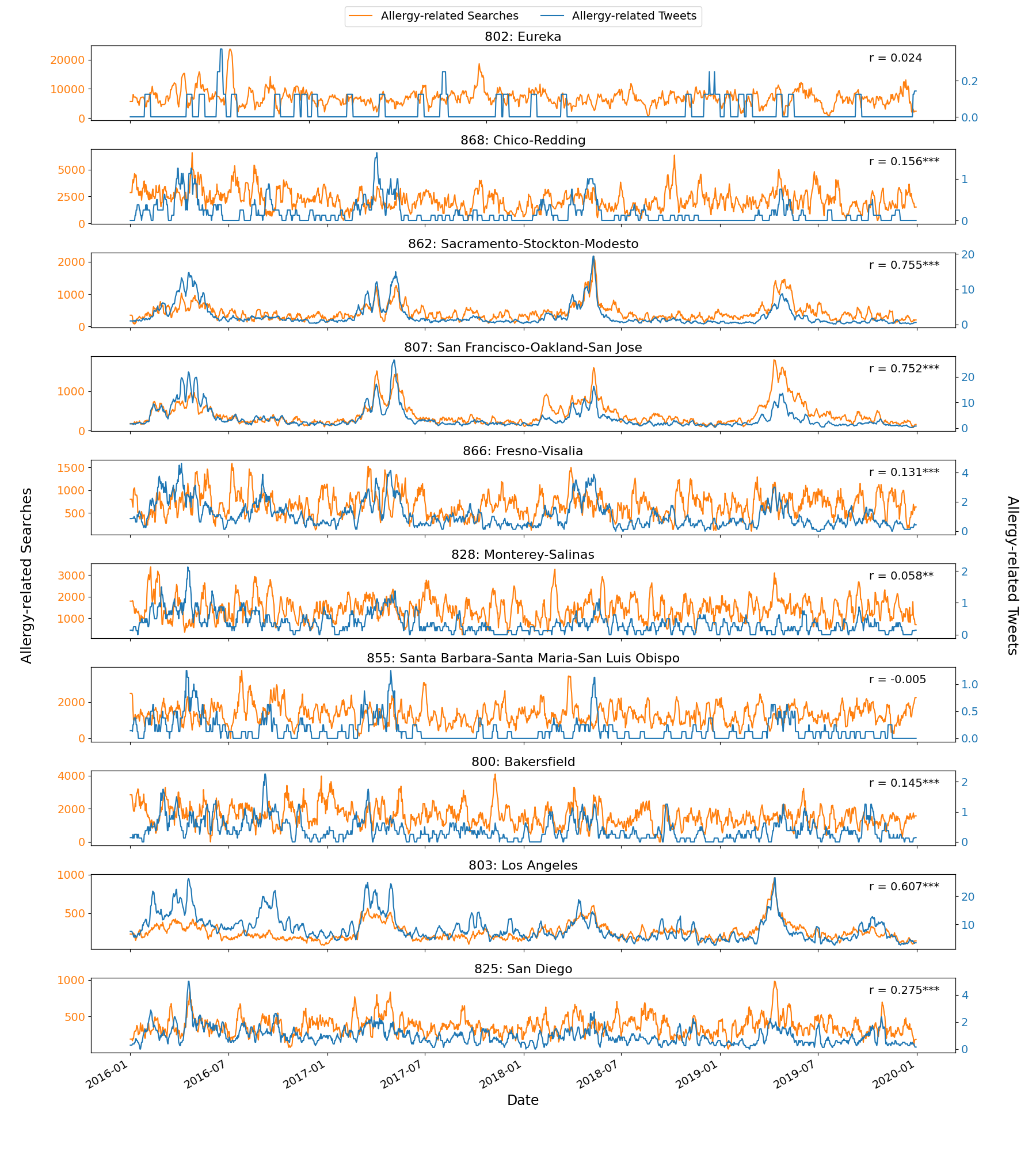


**Fig. S3: Allergy-related searches compared with allergy-related Twitter posts by California DMA**. The relationship between allergy-related Google searches and allergy-related Twitter posts at the DMA level is similar to the relationship between allergy-related Twitter posts and ED records at the county level. However, the mismatch between the areal extent of DMAs and counties likely causes noise in this relationship. Additionally, the relationship between searches and posts is weak in low-population DMAs such as Eureka and Chico-Redding due to the low number of geolocated Twitter posts made in these areas.


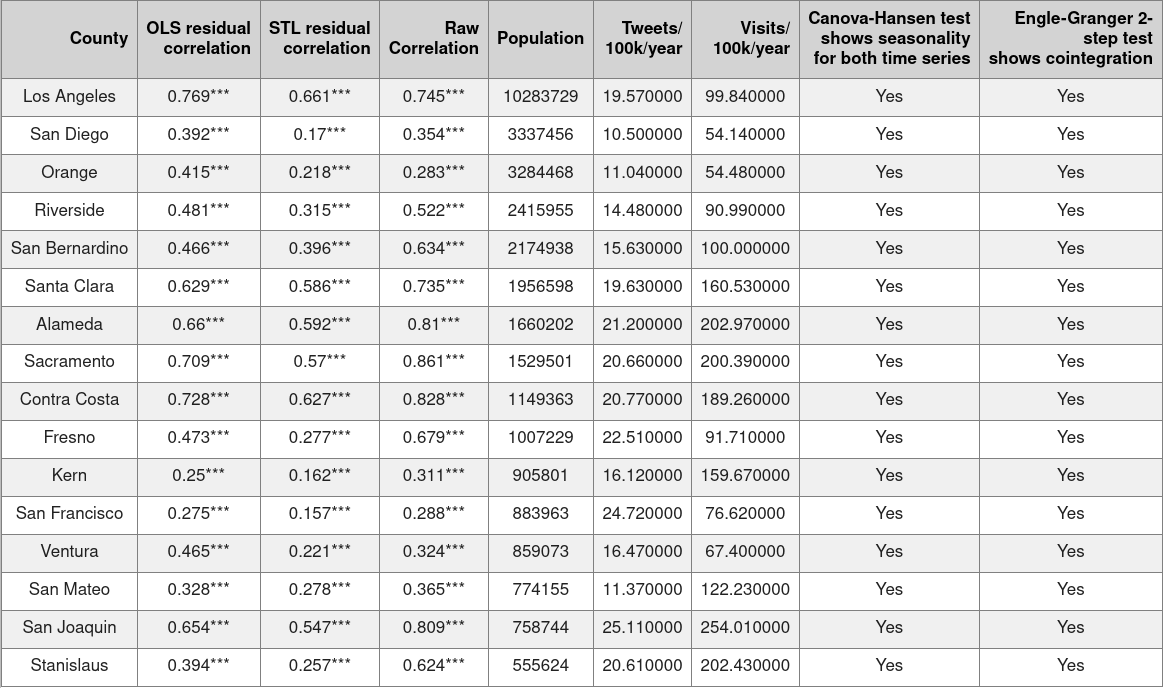


**Table S1: Metrics for the 16 largest California counties, based on Twitter post data.** Canova-Hansen test results indicate that the time series for both allergy-related ED visits and allergy-related Twitter posts have a seasonal unit root. The results for the Engle-Granger 2-step test indicate that the time series are cointegrated with either time series regressed against the other.


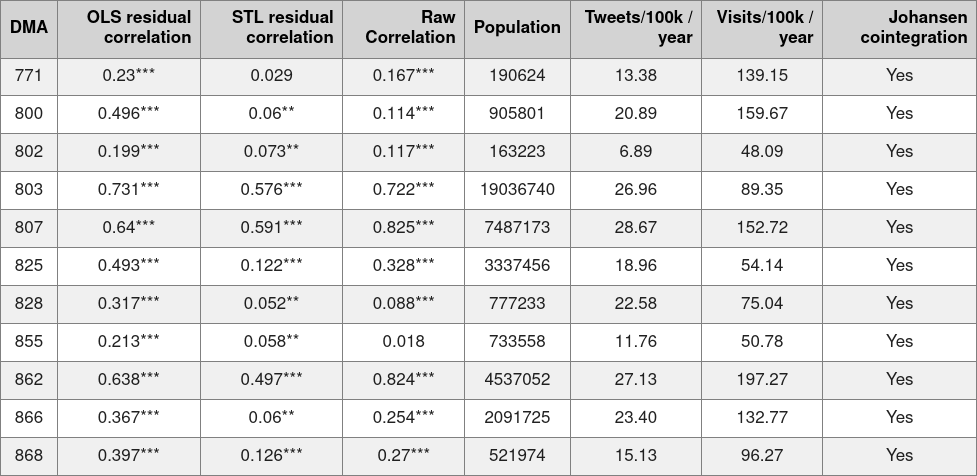


**Table S2: Metrics for California DMAs, based on Google search data.** Johansen cointegration indicates that all three time series - allergy-related Twitter posts aggregated to DMA, allergy-related ED visits aggregated to DMA, and allergy-related Google Trends search probability - are cointegrated to at least rank 1 as per the Johansen cointegration test. Note that DMAs 800 and 862 do not map perfectly to county borders, with 800 only accounting for the western half of Kern County, and 862 not accounting for the eastern half of El Dorado county.


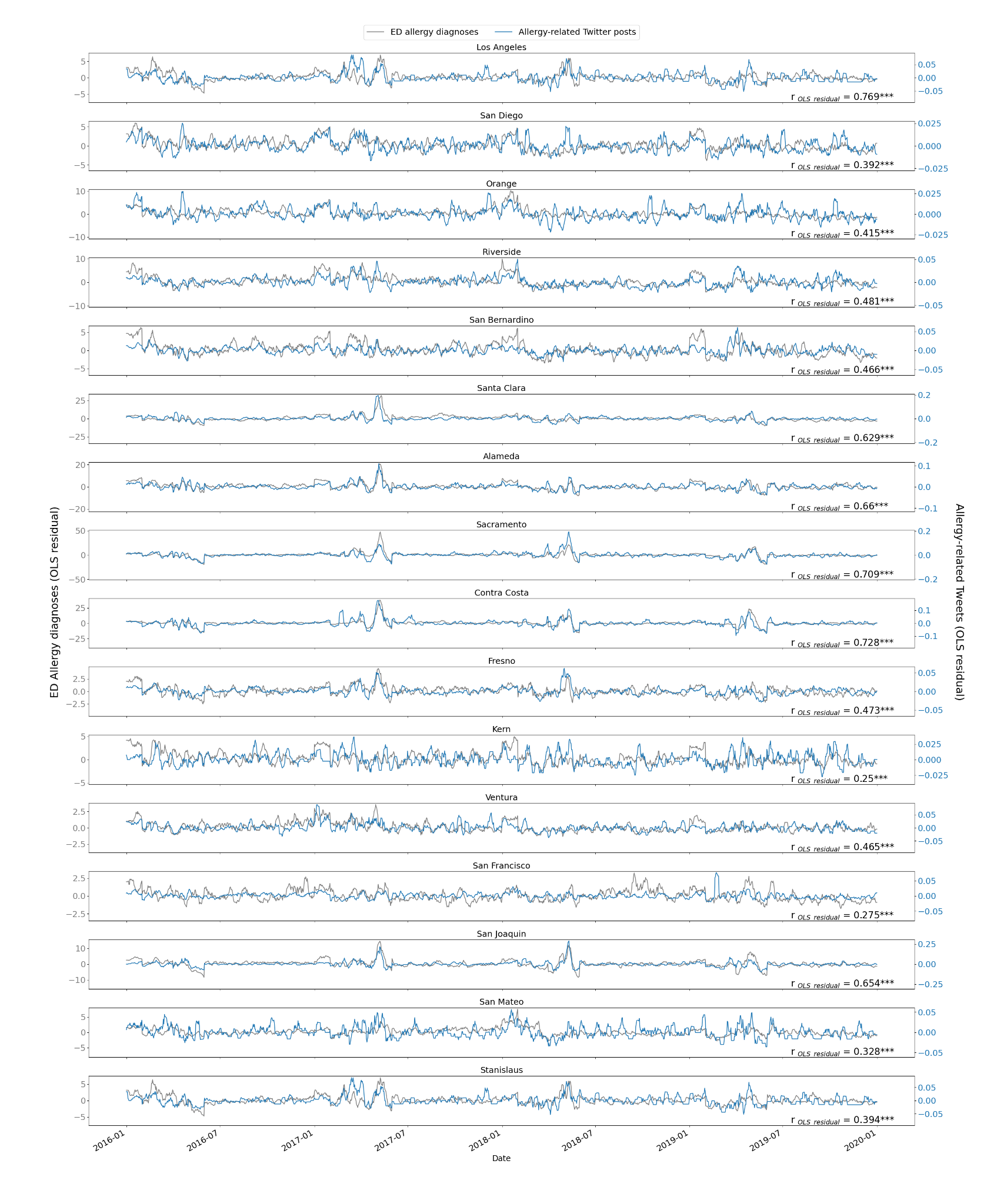


**Fig. S4:** The relationship between the OLS residuals of allergy-related ED codes and allergy-related Twitter posts is typically stronger than that observed among the raw time series. As is the case with both the raw time series and aggregated nationwide data, patterns observed in the time series show similarity between counties in geographic proximity.


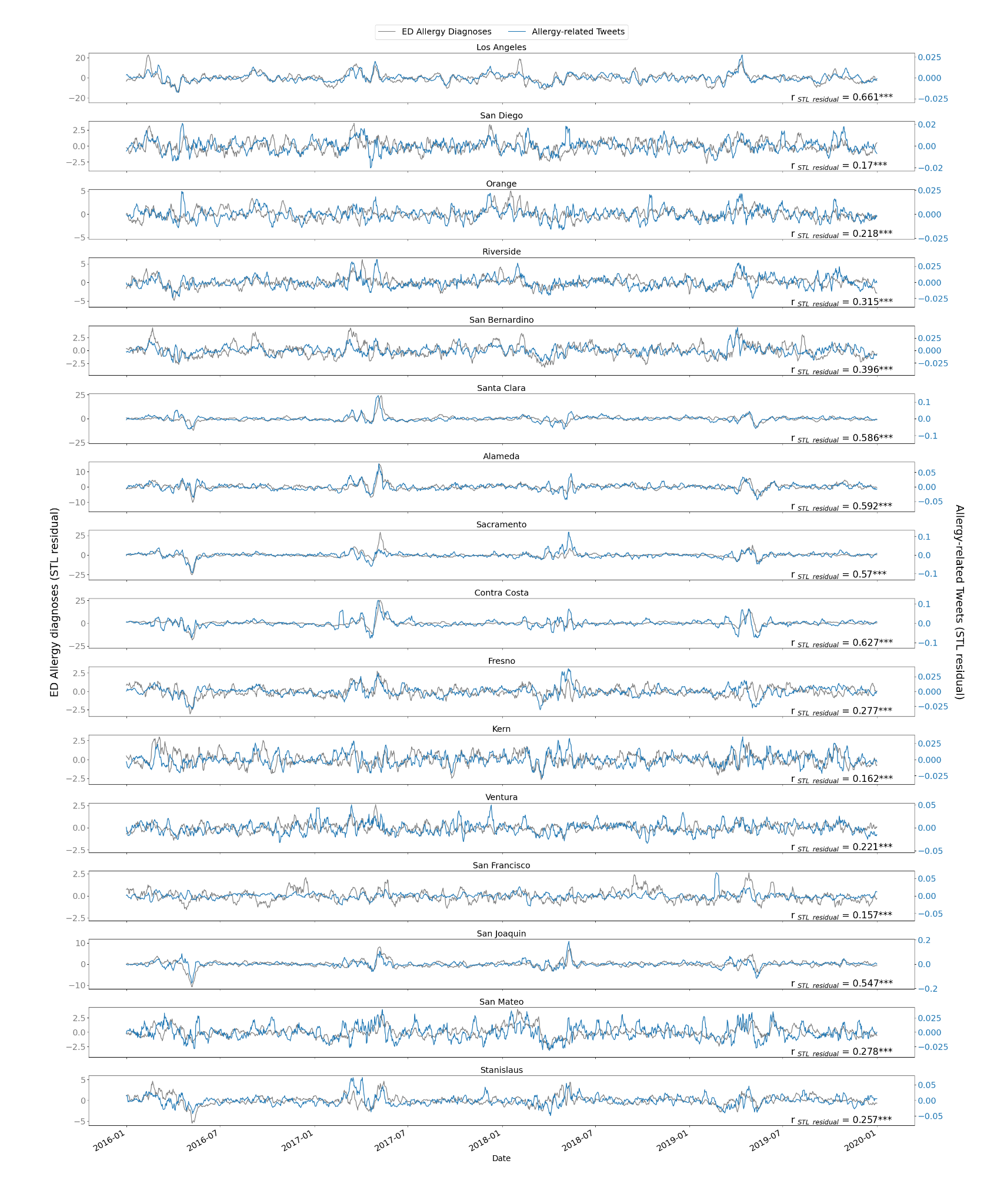
F**ig. S5**: The relationship between the STL residuals of allergy-related ED records and allergy-related Twitter post tends


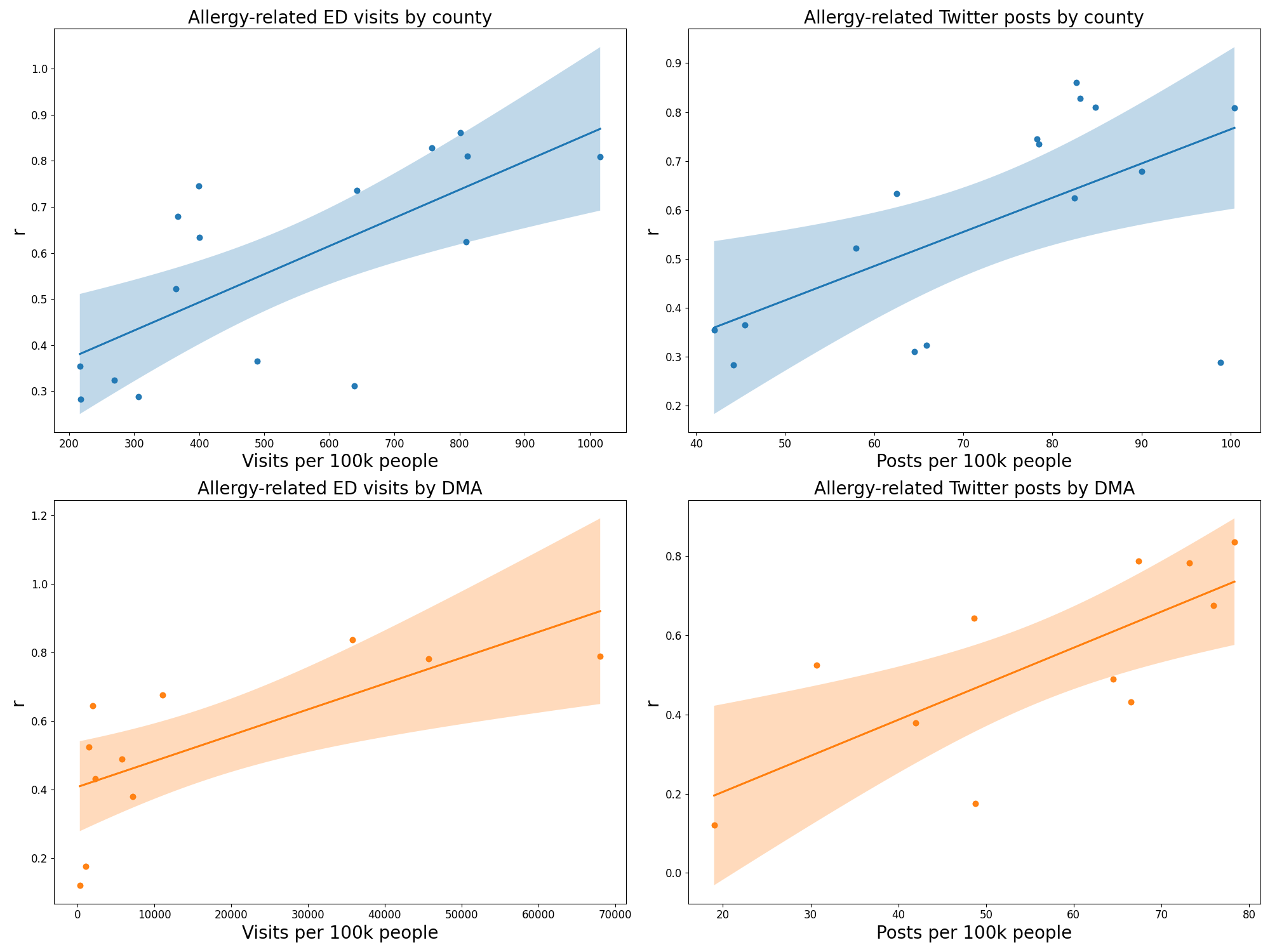


**Fig. S6: Relationship between the areal correlations of ED visits and allergy-related Twitter posts and their population frequency.** There is a positive relationship between both county population and Twitter post volume with the strength of the relationship between the number of allergy-related ED codes and allergy-related twitter posts. This is unsurprising given the relatively small number of ED records and posts collected.


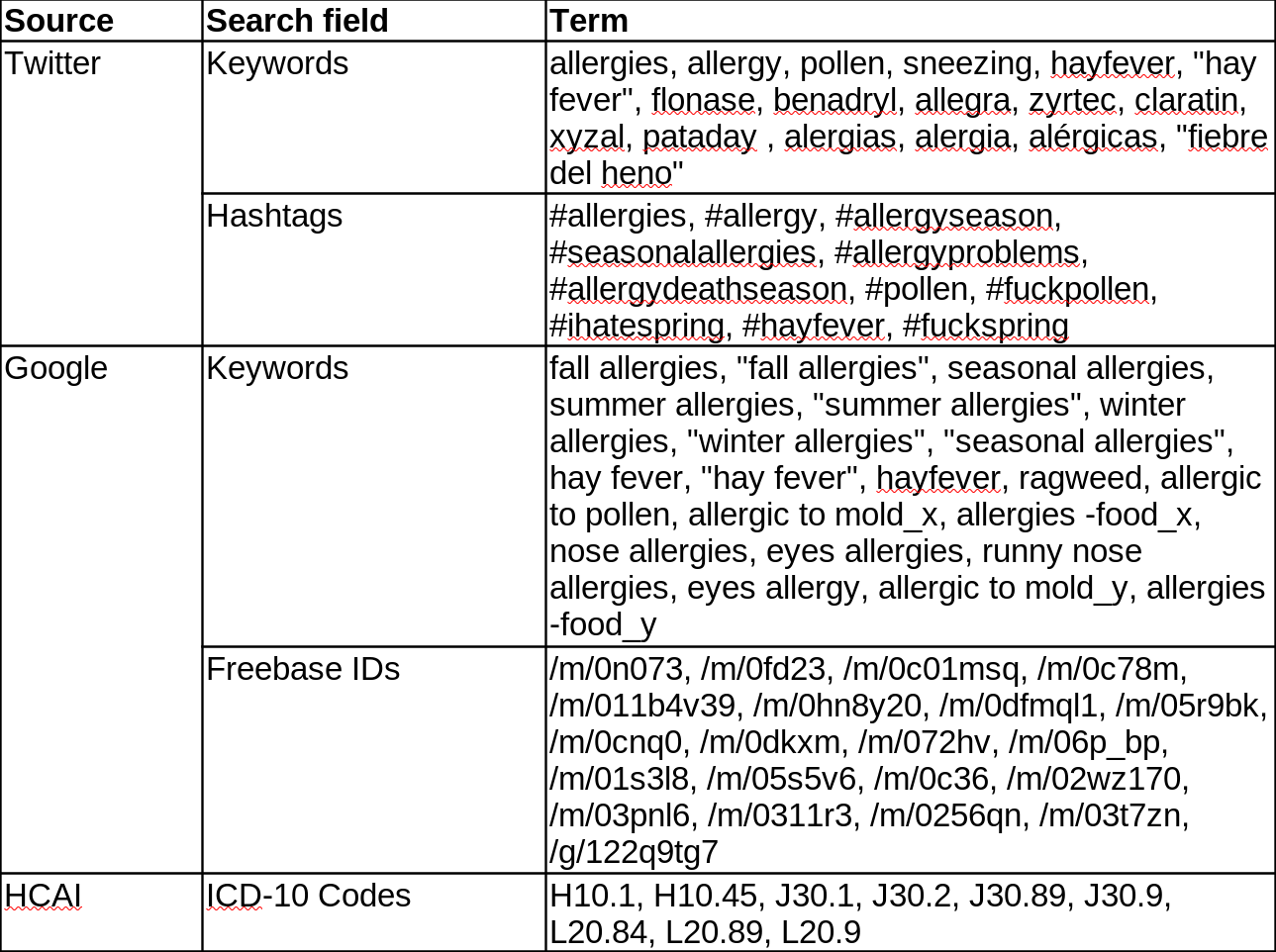


**Table S3: List of terms used to collect data.** Twitter and Google terms were used in their respective API calls to collect data pertaining to allergy-related posts and searches. ICD-10 codes were submitted to HCAI for

**Movie S1: Allergy patterns in the United States according to allergy-related Twitter posts, 2016-2022.** Daily map of allergy-related Twitter posts across the 144 counties in the United States with a population above 500,000, based on the Z-score of Twitter post volume.
